# Cooling methodology: to influence or to control the temperature?

**DOI:** 10.1186/1471-227X-15-S1-A2

**Published:** 2015-06-24

**Authors:** Tommaso Pellis

**Affiliations:** 1 Department of Anesthesia and Intensive Care, Santa Maria degli Angeli Hospital, Pordenone, Italy

## 

The 2010 Guidelines on cardiopulmonary resuscitation and post-resuscitation care, based on the landmark studies on therapeutic hypothermia, state that hypothermia can be induced and maintained with inexpensive means such as simple ice packs and/or wet towels [1]. Indeed there is no method that has proven superior for survival or good neurological outcome. However, this approach comes at the price of an increased burden on nursing staff and greater temperature fluctuations. Also such means do not allow active gradual and controlled rewarming.

While effective in contributing to rapid cooling, simple and inexpensive means may result in greater temperature fluctuations and corresponding modifications in heart rate and electrolyte plasma concentrations. Notably this strategy requires close and constant supervision of nursing staff, a distraction from other important aspects of patient care. Several studies demonstrate that influencing patient temperature will not allow a steady maintenance phase, controlled rewarming and, most importantly, ensuring strict normothermia once rewarming is concluded in patients with evidence of persisting neurological injury [2,3]. In essence, rather than speed it is control that is most desirable. This one of the lessons learned from the recent Target Temperature Management Trial [4]. In this study a less selected population than previous trials was treated at either 33 or 36°C followed by strict normothermia with an automatic feedback device for temperature management. The study demonstrated an extremely high survival rate (approximately 50%) and good neurological outcome regardless of the temperature regimen. Managing temperature at 36°C may overcome many of the contraindications of therapeutic hypothermia at 33°C, but is at the same time more challenging and hardly feasible without automatic feedback devices. Post-rewarming fever is also difficult to manage. Fever is associated with poor outcome. Even if causation has not been proven, normothermia is currently a therapeutic objective of modern post-resuscitation care. Influencing temperature is not enough to ensure strict normothermia. In the Target Temperature Management trial, active temperature management was maintained for a minimum of 72 hours in unconscious patients.

In other fields of application of hypothermia, such as traumatic brain injury treatment and research, protocols dictate prolonged temperature management and extremely slow controlled rewarming based on intracranial pressure.

In conclusion, modern treatment protocols advocate management of temperature, and thus control rather than influence, just like strict management of other vital parameters, is considered a standard of care for the critically ill.

## Financial disclosure

TP has received speaker’s reimbursement from C. R. BARD.

## Declaration

This abstract and supplement was proposed, developed and commissioned by BMC Emergency Medicine and was funded by an educational grant from C. R. BARD, NJ, USA. The published abstract was independently prepared by the author. C. R. BARD had no input into the content.
